# Dilatation of the Heart Consequent upon Teetotalism

**Published:** 1847-01

**Authors:** Richard Chambers

**Affiliations:** Physician to the Essex and Colchester Hospital


*Dilatation of the Heart consequent upon Teetotalism.* By Richard Chambers, M. D., Physician to the Essex and Colchester Hospital.—*Case* I. A gentleman, aged fifty of good constitution, and accustomed to live freely, became a convert to teetotalism. For a period of six months subsequently, there was no perceptible alteration in his state of health ; but about this time he became subject to attacks of nervousness and paroxysms resembling angina, which gradually increased in frequency and intensity up to the time that I visited him. I saw him on August 16th, in consultation with his ordinary attendant. In addition to the above particulars I ascertained, that repeatedly in the course of the night and day, he was subject to attacks of breathlessness, that compelled him to rush to an open window to relieve the sense of impending suffocation. In the intervals he was quite cheerful. He complained of pain in the back of the head ; pulse 84 ; bowels regular; urine copious. On examination of the thorax, (which was extremely well formed,) I found the lungs perfectly sound ; the action of the heart was regular, and its sounds unusually clear, but there was scarcely any impulse. He had been bled the day before, principally at his own request,) but with no relief.

As he had latterly been using a restricted diet, I placed him upon meat daily, with four glasses of good old port, and prescribed five grains of the carbonate of ammonia every four hours, and three grains of the sulphate of quinine thrice a day.

Under this line of treatment he began gradually to amend. At a subsequent period the sulphate of zinc was prescribed for him, and with the alternate use of zinc and quinine, and an increased allowance of wine, his health has been sufficiently restored to enable him to participate in the manly and trying game of cricket.

*Case* II. Henry Taylor, aged 30, a working gardener, applied at the Essex and Colchester Hospital, June 4th, 1846, complaining of headache and general weakness. He was of middle stature, and of a full habit, presenting a slightly bloated appearance. His appetite was good; pulse 78; bowels regular. On stethoscopic examination, the lungs were found to be perfecty healthly ; the heart's action was regular, the sounds clear and healthy, but there was only a very slight impulse. He slept indifferently. For the last six months has been a teetotaller, but denies that he was a drunkard previously.

℞ Pil. saponis; rhei; utrq., gr. iiss. M. Fiat pilula omni nocte sumenda.

℞ Ammon. carb., gr. v.; tinct. camph. comp. dr. ss.; Aquæ, oz. j. M. Sumat ter indies.

15th. His wife applied for medicine for him to-day, and I found that my suspicions as to his intemperate habits were correct.

℞ Pil. saponis, gr. v.; omni nocte. Rept. haust. To have a little gin-and-water after dinner.

This treatment was continued up to the last fortnight, and being free from complaint he discontinued his attendance.

I bring these cases before the association as examples of dilatation of the heart, consequent upon the adoption of teetotalism. The *modus operandi* appears to me to be the yielding of the muscular structure of the heart, in consequence of the less stimulating character of the blood; but, in addition to this, it must not be overlooked, that another element may also be in operation,—I mean an actual increase in the quantity of blood to be circulated, as I have invariably found that a great increase of appetite accompanies teetotalism; not a healthy appetite, but a morbid craving for food, and to oppose which opium is frequently resorted to.

These cases present the principles of the treatment to be adopted, and there is but one caution necessary, and that is, to draw a careful distinction between actual determination of blood to, and merely congestion of blood in, the brain. In the cases just related the headache was referred to the back of the head, and appears to have arisen from venous congestion, but on the stethoscopic induction I wish to place the chief reliance.—*Ibid.*

